# Associations of Fibroblast Growth Factor-23 with Markers of Inflammation, Insulin Resistance and Obesity in Adults

**DOI:** 10.1371/journal.pone.0122885

**Published:** 2015-03-26

**Authors:** Lynae J. Hanks, Krista Casazza, Suzanne E. Judd, Nancy S. Jenny, Orlando M. Gutiérrez

**Affiliations:** 1 Department of Pediatrics, Children’s Hospital of Alabama, University of Alabama at Birmingham, Birmingham, Alabama, United States of America; 2 Department of Biostatistics, University of Alabama at Birmingham, Birmingham, Alabama, United States of America; 3 Pathology and Laboratory Medicine, University of Vermont College of Medicine, Colchester, Vermont, United States of America; 4 Department of Medicine, University of Alabama at Birmingham, Birmingham, Alabama, United States of America; Virgen Macarena University Hospital, School of Medicine, University of Seville, SPAIN

## Abstract

**Introduction:**

Elevated fibroblast growth factor-23 (FGF23) is an established marker of cardiovascular disease. The underlying reason(s) for the rise accompanying cardiovascular health decline are unclear. Prior studies have shown that FGF23 concentrations are associated with markers of inflammation and insulin resistance but they have been limited by a focus on persons with chronic kidney disease (CKD) and lack of race and sex diversity. The objective of this study was to examine the associations of FGF23 and markers of inflammation, insulin resistance, and anthropometrics in a large cohort of community-dwelling adults.

**Methods:**

Associations of FGF23 with markers of inflammation [interleukin-6 (IL-6), IL-10, high sensitivity-CRP (hsCRP)], insulin utilization [resistin, adiponectin, homeostatic model assessment of insulin resistance (HOMA-IR)] and anthropometrics [BMI and waist circumference (WC)] were examined cross-sectionally in a 1,040 participants randomly selected from the Reason for Geographic and Racial Differences in Stroke (REGARDS) Study, a national study of black and white adults ≥45 years. Effect modification by race and CKD status was tested, and stratified models were analyzed accordingly.

**Results:**

Median FGF23 concentration was 69.6 RU/ml (IQR: 53.2, 102.7). Higher quartiles of FGF23 were associated with higher mean concentrations of IL-6, IL-10, hsCRP and resistin (*P*_trend_<0.001 for all). There were no significant differences in HOMA-IR, adiponectin concentrations, BMI, or WC across FGF23 quartiles in the crude analyses. CKD significantly modified the relationships between FGF23 and inflammatory markers, HOMA-IR, BMI and WC (*P ≤ 0*.*01 for all*). In linear regression models adjusted for sociodemographic and clinical variables, FGF23 was positively associated with IL-6, hsCRP, IL-10, HOMA-IR, BMI and WC in individuals without CKD, but not among individuals with CKD. Additionally, FGF23 was positively associated with resistin irrespective of CKD status.

**Conclusions:**

Elevated FGF23 concentrations may be considered a biomarker for decline in metabolic function among individuals with normal kidney function.

## Introduction

Fibroblast growth factor-23 (FGF23) is a hormone primarily produced and secreted by osteocytes that directly regulates phosphorus and vitamin D metabolism [1] [2]. Higher FGF23 has emerged as a risk factor for progressive decline in kidney function and increased cardiovascular disease (CVD) event rates [3–8]. The reasons for these associations are unclear.

FGF23 has been shown to be associated with markers of insulin resistance, dyslipidemia, and visceral adiposity in both children and adults, suggesting that an association of FGF23 with CVD may be in part mediated by cardiometabolic disease [9–11]. However, a causal pathway between circulating concentrations of FGF23 and adverse cardiovascular and renal outcomes remain to be established. Prior studies examining the association of FGF23 with markers of inflammation and insulin resistance were limited by a focus on chronic kidney disease (CKD) populations [12], small sample size and/or lack of race and sex diversity [13,14]. Accordingly, the objective of this study was to examine the associations of FGF23 with markers of inflammation, insulin resistance, and anthropometrics in participants of the Reasons for Geographic and Racial Differences in Stroke (REGARDS) study, a study of community-dwelling black and white adults living throughout the United States (US).

## Materials and Methods

### Study Participants

The Reasons for Geographic and Racial Differences in Stroke (REGARDS) Study is a population-based investigation of stroke incidence in black and white US adults ≥45 years of age. Details of the study design have been reviewed elsewhere [15]. Briefly, participants were recruited from the 48 contiguous US states and the District of Columbia. The study was designed to provide approximately equal representation of men and women, and oversampled blacks and persons living in the “stroke belt/buckle” of the US, both groups who have excess stroke mortality. Trained interviewers conducted computer-assisted telephone interviews to obtain information including participants’ socio-demographics, cardiovascular risk factors, tobacco usage, physical activity, and use of medications. Following this call, an in-home study visit was conducted that included an electrocardiograph (ECG) recording, inventory of medications and collection of blood and urine samples.

Overall, 30,239 black and white adults were enrolled between January 2003 and October 2007 (42% black, 55% women). For this study, we used a subset of REGARDS participants with measures of mineral metabolism (n = 1,104) selected using a stratified random sampling procedure to ensure sufficient participants from high risk categories were represented (e.g., black individuals and older participants), as described previously [16,17]. The REGARDS study protocol was approved by the Institutional Review Boards governing research in human subjects at the participating centers and all participants provided informed consent.

### Methods

Phlebotomy was performed at the participant’s home by trained personnel using standard procedures. Participants were asked to fast for 10–12 hours and samples were shipped overnight on ice to the REGARDS central laboratory as previously described [18,19]. FGF23 was measured using a second-generation C-terminal ELISA (Immutopics, Santa Clara, CA) with inter-assay CVs of 2.3–9.0%. IL-6 was measured by ultra-sensitive ELISA (Quantikine HS Human IL-6 Immunoassay; R&D Systems, Minneapolis, MN); inter-assay CV range of 6.8–7.3%. IL-10 was measured using the Milliplex MAP Human Cardiovascular Disease (CVD) Panel 3 (Millipore Corporation; Billerica, MA) run as a single-plex assay; inter-assay CV range of 8.3–12.1%. High sensitivity CRP was measured by particle enhanced immunonephelometry using the BNII nephelometer (N High Sensitivity CRP; Dade Behring, Deerfield, IL) with inter-assay coefficients of variation (CVs) of 2.1–5.7% [20]. Serum glucose and insulin were measured using the Ortho Vitros 950 IRC Clinical Analyzer (Johnson & Johnson Clinical Diagnostics, Raritan, NJ) and Roche Elecsys 2010 System (Roche Diagnostics, Indianapolis, IN), respectively. Insulin resistance was assessed using the homeostasis model [HOMA-IR = insulin [mg/dL] x glucose [mg/dL] / 405] [21]. Fasting insulin was only obtained in participants without a history diabetes, so calculations of HOMA-IR were only available for those without diabetes (n = 810). Resistin and adiponectin were measured using Human Serum Adipokine Panel A LINCOplex Kit (Linco Research, Inc.; St. Charles, MO). Inter-assay CVs ranges from 8.0–13.2% and 6.1–10.4%, respectively.

Age, race, sex, annual family income, educational attainment, and tobacco and alcohol use history were determined by self-report. Following a standardized protocol, weight, height, and waist circumference were measured during the initial subject examination at the in-home visit. BMI was calculated as weight in kilograms divided by height in meters squared. Waist circumference (in centimeters) was measured during the in-home visit using a tape measure positioned midway between the lowest rib and the iliac crest with the participant standing. Physical activity was assessed through a single question: “How many times per week do you engage in intense physical activity, enough to work up a sweat,” with response options of: none, 1–3 times/week or >4 times/week. History of coronary heart disease (CHD) was defined as having any of the following: evidence of myocardial infarction on the baseline ECG, self-report of a prior history of a cardiac procedure (coronary artery bypass surgery or percutaneous angioplasty), or self-reported history of myocardial infarction. History of stroke was ascertained by self-report. Diabetes was defined as self-reported use of insulin or oral hypoglycemic agents, measured fasting blood glucose concentration of 126 mg/dL or higher, or a measured non-fasting blood glucose concentration of 200 mg/dL or higher.

Serum creatinine was calibrated to an international isotope dilution mass spectroscopic (IDMS)-traceable standard, measured by colorimetric reflectance spectrophotometry. Estimated glomerular filtration rate (eGFR) was calculated using the CKD-EPI equation.[22] Albumin and creatinine were measured in a random spot urine specimen by nephelometry (BN ProSpec Nephelometer, Dade Behring, Marburg, Germany) and Modular-P chemistry analyzer (Roche/Hitachi, Indianapolis, IN), respectively. Spot urine albumin-to-creatinine ratio (UACR) was calculated in mg/g. Prevalent CKD was defined as an eGFR <60 ml/min/1.73m^2^ or a UACR ≥30 mg/g.

### Statistical Analyses

We used a stratified cohort random sample from an existing case cohort within REGARDS. To account for the stratified sampling design of the subcohort, all analyses were weighted by the inverse of the random cohort sampling fraction to weight each subcohort member back to the original cohort [23]. Standard descriptive statistics were used to examine demographic, dietary, clinical and laboratory characteristics of the participants in the overall sample and across quartiles of FGF23 concentrations. Generalized linear models were used to compare energy-adjusted calcium and phosphorus intake by quartile of FGF23. Linear regression models were used to examine the association of FGF23 as the primary independent variable with markers of inflammation (IL-6, hsCRP, IL-10), insulin utilization (resistin, adiponectin, HOMA-IR) and anthropometrics (BMI and waist circumference) as the dependent variables of interest. The initial models were adjusted for age, sex, race, and region of residence. The second model was further adjusted for indices of socioeconomic status, history of diabetes, coronary heart disease, stroke, lifestyle habits (tobacco usage, alcohol consumption, physical activity), plasma calcium and phosphorus, eGFR and UACR. Since FGF23, IL-6, IL-10, resistin, adiponectin, hsCRP and UACR were not normally distributed, we log-transformed these variables for all analyses. We examined effect modification by race and CKD status by testing the significance (*P* < 0.1) of multiplicative interaction terms in the models. Race did not modify any of the relationships, whereas CKD status modified the relationship between FGF23 and each of the inflammatory markers, HOMA-IR, BMI and WC; thus stratified models were analyzed accordingly. A two-tailed P value <0.05 was considered statistically significant for all analyses other than the tests for interaction, in which a P value < 0.1 was considered statistically significant. All analyses were conducted using SAS software version 9.4 (SAS Institute, Cary, NC).

## Results and Discussion

After excluding 64 participants with missing FGF23 concentrations, a total of 1,040 participants were included in the analyzed sample. The mean age of the study sample was 65 ± 0.3 years, 45% were male and 41% were black. Median FGF23 was 69.6 RU/ml (53.2, 102.7). Table 1 presents sociodemographic, behavioral and clinical characteristics in the overall sample and by FGF23 quartile. Higher FGF23 was associated with greater age, female sex, white race, lower household income and education, greater prevalence of CHD, stroke, and diabetes, current smoking, lower alcohol consumption, lower physical activity, higher median UACR and lower eGFR (*P*_trend_ <0.001 for all). The prevalence and severity of CKD were greater in higher quartiles of FGF23 as compared to lower quartiles (*P*_trend_<0.0001).

**Table 1:** Participant characteristics overall and by quartile of fibroblast growth factor-23 level.

Characteristics	Overall	Quartile 1	Quartile 2	Quartile 3	Quartile 4	*P*_*trend*_
		(< 53 RU/ml)	(53–69 RU/ml)	(70–100 RU/ml)	(> 100 RU/ml)	
Weighted N	27,994	6,860	6,971	6,897	7,266	
Age (years)	65.0 (0.3)	62.1 (0.5)	64.0 (0.5)	66.4 (0.6)	67.3 (0.5)	<0.001
Male (%)	45	58	46	46	31	<0.001
Black (%)	41	46	38	45	35	<0.001
Household income < $20,000/yr (%)	16	14	11	18	21	<0.001
Education < high school (%)	12	11	10	12	16	<0.001
Coronary heart disease (%)	17	12	11	15	27	<0.001
Stroke (%)	6	4	6	4	9	<0.001
Diabetes (%)	21	17	15	22	27	<0.001
Dyslipidemia (%)	59	56	60	59	61	<0.001
Current Smoking (%)	14	12	9	14	20	<0.001
Alcohol consumption (%)						<0.001
None (0 drinks/d)	63	56	61	63	70	
Moderate*	33	37	35	33	27	
Heavy **	4	7	4	4	3	
Physical activity frequency (%)***						<0.001
None	34	31	30	31	43	
1–3 times/week	36	40	38	33	34	
>4 times/week	30	29	32	36	23	
UACR (mg/g)	7.1 [4.6, 14.4]	6.4 [4.2, 11.6]	6.6 [4.2, 11.4]	7.2 [4.9, 14.0]	9.3 [4.9, 25.2]	<0.001
eGFR (ml/min/1.73m^2^)	85.9 (0.7)	93.5 (1.1)	88.9 (1.1)	85.3 (1.2)	76.4 (1.5)	<0.001
CKD stage 3a (eGFR 45–59 ml/min/1.73 m^2^), %	6.1	2.2	2.9	6.6	12.2	<0.001
CKD stage 3b (eGFR 30–44 ml/min/1.73 m^2^), %	2.7	0.1	1.9	1.3	7.0	<0.001
CKD stage 4 (eGFR 15–29 ml/min/1.73m^2^), %	0.5	0	0	0.1	1.7	<0.001
CKD stage 5 (eGFR < 15 ml/min/1.73m^2^), %	0.3	0	0	0	1.2	<0.001

Data are given as mean (standard error), median [interquartile range] or frequencies.

Abbreviations: UACR, urinary albumin to creatinine ratio; eGFR, estimated glomerular filtration rate.

* (≤7 and 14 drinks/d for women and men, respectively)

**(>7 and 14 drinks/d for women and men, respectively)

***In answer to “How many times per week do you engage in intense physical activity, enough to work up a sweat?”

**Fig 1:**
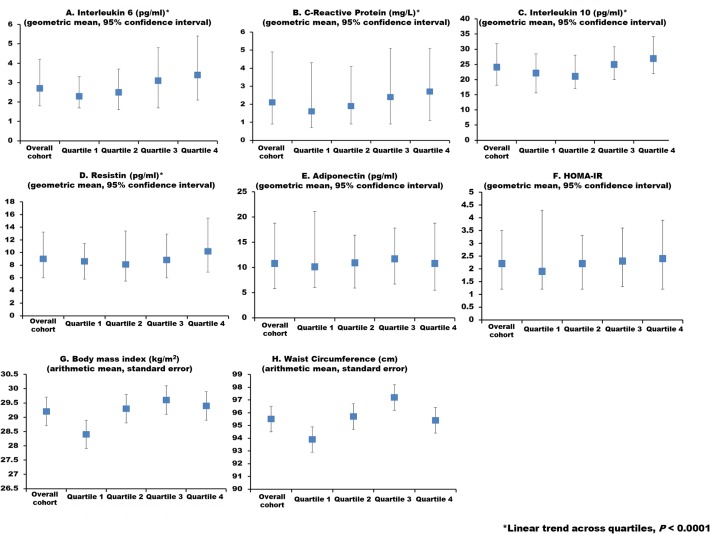
Markers of inflammation, insulin utilization and anthropometrics overall and by quartile of FGF23. The first column represents the overall sample, and the subsequent columns represent FGF23 quartiles 1–4, respectively, in each panel. Values are presented as geometric means, 95% confidence intervals (interleukin-6, high sensitivity C-Reactive protein, interleukin-10, resistin, adiponectin, HOMA-IR)) or mean ± standard deviation (body mass index, waist circumference).

Mean values of markers of inflammation, insulin utilization and anthropometrics are graphed in the overall sample and by FGF23 quartile in Fig 1. Higher quartiles of FGF23 were associated with higher IL-6, hsCRP, IL-10 and resistin (*P* for linear trend <0.001 for all). No statistically significant associations of FGF23 with BMI, waist circumference, adiponectin and HOMA-IR were observed.

Table 2 depicts multivariable-adjusted associations of FGF23 with inflammatory markers (IL-6, IL-10 and hsCRP). CKD significantly modified the relationship between FGF23 and inflammatory markers (*P< 0*.*01 for all*). Therefore, all models were stratified by CKD status. In linear regression models adjusted for age, sex, race, and geographic region of residence, FGF23 was positively associated with IL-6, hsCRP and IL-10 in individuals without CKD but not among individuals with CKD. The magnitude and strength of these associations were qualitatively unchanged after adjustment for sociodemographic, clinical, lifestyle, and laboratory factors.

**Table 2:** Multivariable-adjusted associations between natural log-transformed fibroblast growth factor-23 and natural log-transformed markers of inflammation in the overall sample and by chronic kidney disease (CKD) status.

		Overall			Non-CKD		CKD	
		β	*P*	*P*_*interaction*_	β	*P*	β	*P*
Model 1	IL-6	0.21	<0.001	0.005	0.28	<0.001	0.07	0.35
	hsCRP	0.21	0.002	0.03	0.24	0.02	0.03	0.70
	IL-10	0.21	<0.001	0.08	0.24	0.005	0.09	0.16
Model 2	IL-6	0.18	<0.001	0.02	0.23	<0.001	0.07	0.27
	hsCRP	0.19	0.007	0.04	0.27	0.02	0.02	0.79
	IL-10	0.22	<0.001	0.09	0.31	<0.001	0.003	0.97

Model 1 is adjusted for age, sex, race, region of residence. Model 2 is adjusted for variables in Model 1 plus indices of socioeconomic status, history of diabetes, coronary heart disease, stroke, lifestyle habits (tobacco usage, alcohol consumption, physical activity), serum calcium, serum phosphorus, eGFR and UACR. In models stratified by CKD status, Model 2 was not adjusted for eGFR and UACR. IL-6 = interleukin-6, hsCRP = high-sensitivity c-reactive protein, IL-10 = interleukin-10.

Table 3 presents multivariable-adjusted associations of FGF23 with markers of insulin utilization (resistin, adiponectin, HOMA-IR). FGF23 was positively associated with resistin in models adjusted for age, race, sex and region of residence (*P* < 0.001), and after further adjustment for sociodemographic, clinical, lifestyle, and laboratory factors including kidney function (*P<0*.*001*). Since CKD did not modify the association of FGF23 with resistin, only models examining the full study sample are presented. There were no significant associations of FGF23 with adiponectin in any of the models. In the overall study sample, FGF23 was positively associated with HOMA-IR in the adjusted models. The association of FGF23 with HOMA-IR was modified by CKD (*P* = 0.01); therefore, stratification by CKD status is presented. When stratified by CKD status, the positive association of FGF23 with HOMA-IR was only evident in those without CKD.

**Table 3:** Multivariable-adjusted associations between natural log-transformed fibroblast growth factor-23 and natural log-transformed markers of insulin resistance (resistin; adiponectin; homeostatic model assessment of insulin resistance, HOMA-IR) in the overall sample and by chronic kidney disease (CKD) status (where interaction detected).

		Overall			Non-CKD		CKD	
		β	*P*	*P*_*interaction*_	β	*P*	β	*P*
Model 1	Resistin	0.15	<0.001	0.69				
	Adiponectin	-0.07	0.18	0.81				
	HOMA-IR	0.13	0.01	0.01	0.19	0.008	-0.03	0.72
Model 2	Resistin	0.14	<0.001	0.70				
	Adiponectin	-0.05	0.36	0.71				
	HOMA-IR	0.11	0.03	0.01	0.18	0.01	-0.03	0.59

Model 1 is adjusted for age, sex, race, region of residence. Model 2 is adjusted for variables in Model 1 plus indices of socioeconomic status, history of diabetes (except for analysis of HOMA-IR), coronary heart disease, stroke, lifestyle habits (tobacco usage, alcohol consumption, physical activity), serum calcium, phosphorus, eGFR and ACR. In models stratified by CKD status, Model 2 was not adjusted for eGFR and UACR.

Multivariable-adjusted associations of FGF23 with anthropometrics (BMI and waist circumference) are shown in Table 4. CKD modified the association of FGF23 with both BMI and waist circumference (*P* < 0.01 for both). In adjusted models stratified by CKD status, positive associations of FGF23 with BMI and waist circumference were apparent in individuals without CKD but not among individuals with CKD.

**Table 4:** Multivariable-adjusted associations between natural log-transformed fibroblast growth factor-23 and anthropometrics (BMI and WC, waist circumference) in the overall sample and by chronic kidney disease (CKD) status.

		Overall			Non-CKD		CKD	
		β	*P*	*P*_*interaction*_	β	*P*	β	*P*
Model 1	BMI	0.66	0.04	0.003	1.36	0.005	-0.68	0.19
	WC	1.61	0.04	0.003	3.02	0.004	-1.53	0.28
Model 2	BMI	0.73	0.04	0.007	1.40	0.01	-0.53	0.34
	WC	1.28	0.12	0.01	2.45	0.03	-1.39	0.38

Model 1 is adjusted for age, sex, race, and region of residence. Model 2 is adjusted for indices of socioeconomic status, history of coronary heart disease and/or stroke, lifestyle habits (tobacco usage, alcohol consumption, physical activity), serum calcium, phosphorus, eGFR and UACR. In models stratified by CKD status, Model 2 was not adjusted for eGFR and UACR.

Elevated FGF23 concentrations are associated with greater risk of cardiovascular morbidity and mortality in individuals with CKD [4], and in the general population [24,25]. The reasons for these findings are not clear. In the current study, we found a positive association of FGF23 with key risk factors for CVD, including inflammation, markers of insulin resistance and indices of obesity. Unexpectedly, however, we found that these associations markedly differed by CKD status, such that the magnitude and strength of the association of FGF23 with inflammation and insulin resistance were greater in individuals without vs. those with CKD. Our findings suggest that elevated FGF23 concentrations could serve as a subclinical marker of metabolic perturbations in individuals with normal kidney function.

Other studies have reported an association of FGF23 with inflammatory markers, though the results have been inconsistent. We previously reported that higher FGF23 concentrations were associated with higher IL-6 and vascular cell adhesion molecule 1 (VCAM1) concentrations in 748 participants of the Health Professionals Follow-Up Study [13]. Importantly, this prior study was limited by the inclusion of only men, most of whom were of white race, limiting the generalizability of the findings. The findings of the current study suggest that the association of higher FGF23 with markers of inflammation extend to women and individuals of black race. In 3,879 Chronic Renal Insufficiency Cohort (CRIC) Study participants, FGF23 was positively associated with inflammatory markers (i.e., IL-6, CRP, tumor necrosis factor-α, and fibrinogen) independently of potential confounders, including kidney function [12]. Similarly, a study of individuals with mild to moderate CKD found a positive association of FGF23 with CRP [26]. This is in contrast to our findings in which we found no associations of FGF23 with IL-6, IL-10 and hsCRP in multivariable adjusted analyses among REGARDS participants with CKD. The reasons for these differences are unclear, but may be due to differences in the demographic characteristics of the patient populations. In addition, given that the number of individuals with CKD was substantially lower in the current study as compared to prior studies [12,26], it is possible that we were underpowered to detect an association of FGF23 with inflammation in CKD.

The possible mechanisms for an association of FGF23 with inflammatory markers are largely unclear, though we can speculate as to several possibilities. A prior comparative genome-wide analysis of gene expression profiles in mice with progressive kidney disease found that FGF23 expression was correlated with markers of chronic inflammation [27], suggesting potential interactions between FGF23 and inflammatory regulatory networks. Higher FGF23 could potentially activate the inflammatory cascade by suppressing the synthesis of 1,25-dihydroxyvitamin D [28–30], a potent inhibitor of inflammatory cytokine secretion [31]. It is also possible that FGF23 may be a suitable indirect marker of disease processes associated with inflammation, like kidney disease. Thus, FGF23 concentration may not be in direct association with higher inflammation. The possible correlation of inflammatory markers other than IL-6, IL-10 and CRP with FGF23 in CKD and non-CKD populations should be studied further.

FGF23 may also be a marker of diabetes progression or may increase with diabetes-related complications [32]. We found that the surrogate marker of insulin resistance, HOMA-IR and resistin, an adipokine that is up-regulated in the setting of insulin resistance [33], were positively associated with FGF23. The observed association of FGF23 with HOMA-IR in our cohort was attenuated among individuals with CKD, but remained among those without CKD. While limited, particularly in adults without CKD, this is in agreement with other reports. In 133 non-CKD male patients, insulin sensitivity independently contributed to the variance in FGF23 levels [34]. In the same study, a positive association of FGF23 with HOMA-IR was reported in a separate cohort of 314 men and women, and a parallel decrease in HOMA-IR and FGF23 levels after weight loss was observed in 10 Caucasian obese men [34]. In contrast, Mirza and colleagues did not find an independent association of FGF23 with HOMA-IR in a pooled analysis of the Osteoporotic Fractures in Men and the Prospective Investigation of the Vasculature in Uppsala Seniors studies [14]. A small study has reported an inverse association between FGF23 and HOMA-IR in obese adolescents [9]. In CKD patients with insulin resistance, Garland et al recently reported higher FGF23 level, which was not accounted for by other metabolic syndrome components [11]. Further study on variations in FGF23 concentrations in individuals with impaired insulin sensitivity may provide additional insight.

Animal models have provided insight on potential mechanisms underlying the relation of FGF23 to adiposity and insulin resistance. FGF23^-/-^ mice have a complex phenotype characterized by severe mineral metabolism impairments, shortened lifespan, growth retardation, dysregulated organ development, together with hypoglycemia and profoundly increased peripheral insulin sensitivity. FGF23-ablated young mice (four weeks of age) are characterized by hypoglycemia and improved glucose tolerance due to profoundly improved peripheral insulin sensitivity, a premature aging-like phenotype, and almost complete absence of subcutaneous fat [35]. Other recent studies conducted in mice indicate that insulin signaling disturbances in the genetically ablated FGF23 animals are mediated by vitamin D [27,36]. Speculatively, circulating FGF23 may modulate the binding of Klotho, thereby indirectly influencing insulin signaling. The direct translation of this to humans has not been fully elucidated and may differ by metabolic health status [10,36]. Together, these data support important links between FGF23, adiposity and insulin sensitivity.

Resistin, which was initially considered as a determinant of the emergence of insulin resistance in obesity [37], has appeared as an important link between obesity and inflammatory processes. A growing body of evidence has emerged that resistin is an inflammatory biomarker and potential mediator of obesity-associated diseases in humans. Resistin is expressed primarily by macrophages in the visceral adipose tissue [38], and levels have been shown to be elevated in sera of CKD patients [39–41]. Therefore, it is interesting that higher FGF23 was associated with higher concentrations of resistin. When coupled with the finding that FGF23 was associated with higher BMI and waist circumference, these findings add to the growing body of evidence supporting physiological links between FGF23 and obesity [13,14]. However, the mechanisms which link FGF23 with insulin resistance and obesity in the general population and individuals with impaired kidney function remain to be defined.

Our study has several notable strengths. The relatively large sample of white and black community-dwelling adults utilized in this study provides one of the most comprehensive examinations of FGF23 with markers of inflammation, insulin utilization and obesity in the general population. Our study also had some limitations. The single measurement of the blood values used in the analysis prevents inference on causality. Furthermore, the proportion of participants with early and later stages of CKD (eGFR < 30 ml/min 1.73 m^2^) was relatively low in this cohort, making the results among individuals with CKD most applicable to individuals with mild to moderate stages of disease. Also, we only had a single measurement of UACR and eGFR to account for the contribution of kidney function as a potential confounder, which may have led to exposure misclassification for some study participants. BMI as a measure of obesity cannot distinguish between fat and lean tissue, preventing a potential mechanistic explanation of race-related differences. Similarly, waist circumference may reflect varying levels of abdominal visceral fat, particularly among older populations [42,43]. In addition, we acknowledge that there are a number of potentially relevant confounders that have been reported to contribute to the inflammatory response and progression of metabolic disease (e.g., leptin). We did not have leptin measurements, and therefore were unable to examine the association of FGF23 with leptin. There were also missing or incomplete data on other potentially relevant confounders such as dietary intake.

## Conclusions

Our findings suggest independent associations of FGF23 with insulin resistance, inflammation, and obesity, particularly among individuals with normal kidney function. The progressive increase in circulation of FGF23 prior to overt disease may offer a useful estimation of advancing decline in metabolic health. Thus, these findings warrant consideration of FGF23 as a subclinical marker of metabolic perturbations.

## References

[1] Liu S, Quarles LD. How fibroblast growth factor 23 works. J Am Soc Nephrol. 2007 Jun 18: 1637–1647. 17494882 10.1681/ASN.2007010068

[2] Juppner H, Wolf M, Salusky IB. FGF-23: More than a regulator of renal phosphate handling? J Bone Miner Res. 2010 Oct 25: 2091–2097. 10.1002/jbmr.170 20593414 PMC3153315

[3] Fliser D, Kollerits B, Neyer U, Ankerst DP, Lhotta K, Lingenhel A, et al Fibroblast growth factor 23 (FGF23) predicts progression of chronic kidney disease: the Mild to Moderate Kidney Disease (MMKD) Study. J Am Soc Nephrol. 2007 Sep 18: 2600–2608. 17656479 10.1681/ASN.2006080936

[4] Gutierrez OM, Mannstadt M, Isakova T, Rauh-Hain JA, Tamez H, Shah A, et al Fibroblast growth factor 23 and mortality among patients undergoing hemodialysis. N Engl J Med. 2008 Aug 7; 359: 584–592. 10.1056/NEJMoa0706130 18687639 PMC2890264

[5] Isakova T, Xie H, Yang W, Xie D, Anderson AH, Scialla J, et al Fibroblast growth factor 23 and risks of mortality and end-stage renal disease in patients with chronic kidney disease. JAMA. 2011 Jun 15; 305: 2432–2439. 10.1001/jama.2011.826 21673295 PMC3124770

[6] Jean G, Terrat JC, Vanel T, Hurot JM, Lorriaux C, Mayor B, et al High levels of serum fibroblast growth factor (FGF)-23 are associated with increased mortality in long haemodialysis patients. Nephrol Dial Transplant. 2009 Sep 24: 2792–2796. 10.1093/ndt/gfp191 19395730

[7] Parker BD, Schurgers LJ, Brandenburg VM, Christenson RH, Vermeer C, Kettler M, et al The associations of fibroblast growth factor 23 and uncarboxylated matrix Gla protein with mortality in coronary artery disease: the Heart and Soul Study. Ann Intern Med. 2010 May 18; 152: 640–648. 10.7326/0003-4819-152-10-201005180-00004 20479029 PMC3079370

[8] Seiler S, Heine GH, Fliser D. Clinical relevance of FGF-23 in chronic kidney disease. Kidney Int. 2009 Dec Suppl: S34–42. 10.1038/ki.2009.405 19946326

[9] Wojcik M, Dolezal-Oltarzewska K, Janus D, Drozdz D, Sztefko K, Starzyk JB. FGF23 contributes to insulin sensitivity in obese adolescents—preliminary results. Clin Endocrinol. 2012 Oct (Oxf) 77: 537–540. 10.1111/j.1365-2265.2011.04299.x 22103239

[10] Wojcik M, Janus D, Dolezal-Oltarzewska K, Drozdz D, Sztefko K, Starzyk JB. The association of FGF23 levels in obese adolescents with insulin sensitivity. J Pediatr Endocrinol Metab. 2012 25: 687–690. 23155694 10.1515/jpem-2012-0064

[11] Garland JS, Holden RM, Ross R, Adams MA, Nolan RL, Hopman WM, et al Insulin resistance is associated with Fibroblast Growth Factor-23 in stage 3–5 chronic kidney disease patients. J Diabetes Complications. 2014 Jan-Feb 28: 61–65. 10.1016/j.jdiacomp.2013.09.004 24125760

[12] Munoz Mendoza J, Isakova T, Ricardo AC, Xie H, Navaneethan SD, Anderson AH, et al Fibroblast growth factor 23 and Inflammation in CKD. Clin J Am Soc Nephrol. 2012 Jul 7: 1155–1162. 10.2215/CJN.13281211 22554719 PMC3386678

[13] Gutierrez OM, Wolf M, Taylor EN. Fibroblast growth factor 23, cardiovascular disease risk factors, and phosphorus intake in the health professionals follow-up study. Clin J Am Soc Nephrol. 2011 Dec 6: 2871–2878. 10.2215/CJN.02740311 22034506 PMC3255372

[14] Mirza MA, Alsio J, Hammarstedt A, Erben RG, Michaelsson K, Tivesten A, et al Circulating fibroblast growth factor-23 is associated with fat mass and dyslipidemia in two independent cohorts of elderly individuals. Arterioscler Thromb Vasc Biol. 2011 Jan 31: 219–227. 10.1161/ATVBAHA.110.214619 20966399

[15] Howard VJ, Cushman M, Pulley L, Gomez CR, Go RC, Prineas RJ, et al The reasons for geographic and racial differences in stroke study: objectives and design. Neuroepidemiology. 2005 25: 135–143. 15990444 10.1159/000086678

[16] Zakai NA, Judd SE, Alexander K, McClure LA, Kissela BM, Howard G, et al ABO blood type and stroke risk: the REasons for Geographic And Racial Differences in Stroke Study. J Thromb Haemost. 2014 Apr 12: 564–570. 10.1111/jth.12507 24444093 PMC4913462

[17] Alexander KS, Zakai NA, Gillett S, McClure LA, Wadley V, Unverzagt F, et al ABO blood type, factor VIII, and incident cognitive impairment in the REGARDS cohort. Neurology. 2014 Sep 30; 83: 1271–1276. 10.1212/WNL.0000000000000844 25209581 PMC4180487

[18] Cushman M, McClure LA, Howard VJ, Jenny NS, Lakoski SG, Howard G. Implications of increased C-reactive protein for cardiovascular risk stratification in black and white men and women in the US. Clin Chem. 2009 Sep 55: 1627–1636. 10.1373/clinchem.2008.122093 19643839 PMC2810186

[19] Gillett SR, Boyle RH, Zakai NA, McClure LA, Jenny NS, Cushman M. Validating laboratory results in a national observational cohort study without field centers: The Reasons for Geographic and Racial Differences in Stroke cohort. Clin Biochem. 2014 Aug 14. 10.1016/j.clinbiochem.2014.08.003 PMC5038129 25130959

[20] Suzuki T, Voeks J, Zakai NA, Jenny NS, Brown TM, Safford MM, et al Metabolic Syndrome, C-Reactive Protein, and Mortality in U.S. Blacks and Whites: The Reasons for Geographic and Racial Differences in Stroke (REGARDS) Study. Diabetes Care. 2014 Aug 37: 2284–2290. 10.2337/dc13-2059 24879838 PMC4113170

[21] Matthews DR, Hosker JP, Rudenski AS, Naylor BA, Treacher DF, Turner RC. Homeostasis model assessment: insulin resistance and beta-cell function from fasting plasma glucose and insulin concentrations in man. Diabetologia. 1985 Jul 28: 412–419. 3899825 10.1007/BF00280883

[22] Levey AS, Stevens LA, Schmid CH, Zhang YL, Castro AF 3rd, Feldman HI, et al A new equation to estimate glomerular filtration rate. Ann Intern Med. 2009 May 5; 150: 604–612. 19414839 10.7326/0003-4819-150-9-200905050-00006 PMC2763564

[23] Barlow WE, Ichikawa L, Rosner D, Izumi S. Analysis of case-cohort designs. J Clin Epidemiol. 1999 Dec 52: 1165–1172. 10580779 10.1016/s0895-4356(99)00102-x

[24] Mirza MA, Larsson A, Melhus H, Lind L, Larsson TE. Serum intact FGF23 associate with left ventricular mass, hypertrophy and geometry in an elderly population. Atherosclerosis. 2009 Dec 207: 546–551. 10.1016/j.atherosclerosis.2009.05.013 19524924

[25] Donate-Correa J, Muros-de-Fuentes M, Mora-Fernandez C, Navarro-Gonzalez JF. FGF23/Klotho axis: phosphorus, mineral metabolism and beyond. Cytokine Growth Factor Rev. 2012 Feb-Apr 23: 37–46. 10.1016/j.cytogfr.2012.01.004 22360923

[26] Manghat P, Fraser WD, Wierzbicki AS, Fogelman I, Goldsmith DJ, Hampson G. Fibroblast growth factor-23 is associated with C-reactive protein, serum phosphate and bone mineral density in chronic kidney disease. Osteoporos Int. 2010 Nov 21: 1853–1861. 10.1007/s00198-009-1142-4 20012018

[27] Dai B, David V, Martin A, Huang J, Li H, Jiao Y, et al A comparative transcriptome analysis identifying FGF23 regulated genes in the kidney of a mouse CKD model. PLoS One. 2012 7: e44161 10.1371/journal.pone.0044161 22970174 PMC3435395

[28] Hasegawa H, Nagano N, Urakawa I, Yamazaki Y, Iijima K, Fujita T. Direct evidence for a causative role of FGF23 in the abnormal renal phosphate handling and vitamin D metabolism in rats with early-stage chronic kidney disease. Kidney Int. 2010 78: 975–980. 10.1038/ki.2010.313 20844473

[29] Shimada T, Urakawa I, Isakova T, Yamazaki Y, Epstein M, Wesseling-Perry K, et al Circulating fibroblast growth factor 23 in patients with end-stage renal disease treated by peritoneal dialysis is intact and biologically active. J Clin Endocrinol Metab. 2010 Feb 95: 578–585. 10.1210/jc.2009-1603 19965919 PMC2840849

[30] Kovesdy CP, Quarles LD. Fibroblast growth factor-23: what we know, what we don't know, and what we need to know. Nephrol Dial Transplant. 2013 Sep 28: 2228–2236. 10.1093/ndt/gft065 23625971 PMC3769978

[31] Gao D, Trayhurn P, Bing C (2013) 1,25-Dihydroxyvitamin D3 inhibits the cytokine-induced secretion of MCP-1 and reduces monocyte recruitment by human preadipocytes. Int J Obes (Lond) 37: 357–365. 10.1038/ijo.2012.53 22508334 PMC3428854

[32] Wahl P, Xie H, Scialla J, Anderson CA, Bellovich K, Brecklin C, et al Earlier onset and greater severity of disordered mineral metabolism in diabetic patients with chronic kidney disease. Diabetes Care. 2012 May 35: 994–1001. 10.2337/dc11-2235 22446176 PMC3329844

[33] Chen BH, Song Y, Ding EL, Roberts CK, Manson JE, Rifai N, et al Circulating levels of resistin and risk of type 2 diabetes in men and women: results from two prospective cohorts. Diabetes Care. 2009 Feb 32: 329–334. 10.2337/dc08-1625 18957529 PMC2628703

[34] Fernandez-Real JM, Puig J, Serrano M, Sabater M, Rubio A, Moreno-Navarrete JM, et al Iron and obesity status-associated insulin resistance influence circulating fibroblast-growth factor-23 concentrations. PLoS One. 2013 8: e58961 10.1371/journal.pone.0058961 23555610 PMC3605441

[35] Hesse M, Frohlich LF, Zeitz U, Lanske B, Erben RG. Ablation of vitamin D signaling rescues bone, mineral, and glucose homeostasis in Fgf-23 deficient mice. Matrix Biol. 2007 Mar 26: 75–84. 17123805 10.1016/j.matbio.2006.10.003

[36] Streicher C, Zeitz U, Andrukhova O, Rupprecht A, Pohl E, Larsson TE, et al Long-term Fgf23 deficiency does not influence aging, glucose homeostasis, or fat metabolism in mice with a nonfunctioning vitamin D receptor. Endocrinology. 2012 Apr 153: 1795–1805. 10.1210/en.2011-1878 22294750 PMC3320267

[37] Steppan CM, Bailey ST, Bhat S, Brown EJ, Banerjee RR, Wright CM, et al The hormone resistin links obesity to diabetes. Nature. 2001 Jan 18; 409: 307–312. 11201732 10.1038/35053000

[38] Curat CA, Wegner V, Sengenes C, Miranville A, Tonus C, Busse R, et al Macrophages in human visceral adipose tissue: increased accumulation in obesity and a source of resistin and visfatin. Diabetologia. 2006 Apr 49: 744–747. 16496121 10.1007/s00125-006-0173-z

[39] Vanholder R, De Smet R, Glorieux G, Argiles A, Baurmeister U, Brunet P, et al Review on uremic toxins: classification, concentration, and interindividual variability. Kidney Int. 2003 May 63: 1934–1943. 12675874 10.1046/j.1523-1755.2003.00924.x

[40] Diez JJ, Iglesias P, Fernandez-Reyes MJ, Aguilera A, Bajo MA, Alvarez-Fidalgo P, et al Serum concentrations of leptin, adiponectin and resistin, and their relationship with cardiovascular disease in patients with end-stage renal disease. Clin Endocrinol. 2005 Feb (Oxf) 62: 242–249. 15670203 10.1111/j.1365-2265.2005.02207.x

[41] Widjaja A, Kielstein JT, Horn R, von zur Muhlen A, Kliem V, Brabant G. Free serum leptin but not bound leptin concentrations are elevated in patients with end-stage renal disease. Nephrol Dial Transplant. 2000 Jun 15: 846–850. 10831639 10.1093/ndt/15.6.846

[42] Harris TB, Visser M, Everhart J, Cauley J, Tylavsky F, Fuerst T, et al Waist circumference and sagittal diameter reflect total body fat better than visceral fat in older men and women. The Health, Aging and Body Composition Study. Ann N Y Acad Sci. 2000 Jun 904: 462–473. 10865790 10.1111/j.1749-6632.2000.tb06501.x

[43] Hill JO, Sidney S, Lewis CE, Tolan K, Scherzinger AL, Stamm ER. Racial differences in amounts of visceral adipose tissue in young adults: the CARDIA (Coronary Artery Risk Development in Young Adults) study. Am J Clin Nutr. 1999 Mar 69: 381–387. 10075320 10.1093/ajcn/69.3.381

